# Visibility in Varying Light Conditions During Simulated Neonatal Transport

**DOI:** 10.7759/cureus.81892

**Published:** 2025-04-08

**Authors:** Andia Pouresfandiary Cham, Matthew W Cook, John Feltner, Rachel A Umoren

**Affiliations:** 1 Bioengineering, University of Washington, Seattle, USA; 2 Pediatrics, University of Washington School of Medicine, Seattle, USA; 3 Pediatrics/Neonatology, Seattle Children's Hospital, Seattle, USA

**Keywords:** light, neonatal, pediatrics, safety, simulation, transport, visibility

## Abstract

Background and objectives: During neonatal transport, specialized pediatric transport teams closely monitor the status of critically ill newborns. Teletransport applications require an appropriate light intensity for the visibility of the neonate, but this must be balanced with safety for sensitive eyes. This simulation study was conducted to determine the amount of light needed to view a neonatal manikin during transport.

Methods: The potential light exposure to the eyes of the neonate was measured using light bars and a photometer in a simulated transport setting with a newborn manikin in a transport incubator. Twelve videos depicting the visibility of the manikin in the incubator were recorded in controlled amounts of light and viewed by experienced medical control physicians (MCPs). Eight neonatologist MCPs participated in a survey.

Results: Based on MCP ratings, the location of the light source for optimal viewing was along the inside upper long edge above the access door. Analysis of the range of desired light intensity showed that the amount of light that reached the patient’s eyes (1 to 22.5 lux) was significantly lower than the intensity at the light source (1 to 351 lux) and did not increase linearly with the increasing light intensity.

Conclusion: There was variability in the range of desired light intensity for remote patient monitoring during neonatal transport. More studies on visibility and safety are needed to inform approaches to remote patient monitoring during transports.

## Introduction

Each year, 68,000 critically ill newborns in need of medical treatment are transported over long distances to receive care [1]. Neonates are transported by land or air in incubators with medical devices monitoring their vital signs during transport. In the Pacific Northwest region of the U.S., care during transport is provided by specially trained healthcare professionals including transport nurses and respiratory therapists, with a remotely located neonatologist acting as a medical control physician (MCP) at a level four NICU with the central communications center for the region. 

Despite having highly trained professionals overseeing transport, adverse events occur in 22% of all pediatric critical care ambulance transports [2]. One-third of these adverse events are due to incomplete and inaccurate information [3]. Others are due to other factors such as improper assessment of urgency or selection of equipment [4]. Teletransport is the real-time monitoring of the neonatal patient by an MCP during transport by using video cameras and other electronic communication tools [5]. The application of telemedicine to neonatal care during transport may be beneficial for MCPs to obtain accurate, complete, and timely information, but may also be limited by the poor lighting conditions inside the transport incubator. 

Lighting in the transport vehicle can be limited, making it difficult to see the patient’s physical condition. Covers are also placed over the incubator during transports to help insulate it, as well as to provide protection and comfort from outside light. Some neonatal patients, specifically micro-preemies, have light-sensitive eyes that must be protected. 

For telemedicine to be feasible during neonatal transport, the amount of light reaching a neonate’s eyes should not be harmful, while being sufficient enough for the medical care provider to have clear visibility of the neonate. In some cases, flashlights are used briefly when needed to view the patient; however, this is not always the best way to increase visibility as the light is too intense for continuous monitoring [6]. Adequate lighting in the neonatal incubator during transport will facilitate the optimal viewing of the baby and support informed medical decisions. The goal of this pilot study is to determine the range of light intensity required for a remote MCP to view the patient on video.

This article was previously presented as a poster at the 2023 University of Washington Undergraduate Research Symposium on May 19, 2023.

## Materials and methods

Study design 

This observational study was conducted from March to June 2022 at a regional referral children’s hospital (Seattle Children's Hospital, Seattle, USA) in the Pacific Northwest.

Ethical Considerations

This study was approved by the University of Washington Institutional Review Board, with approval number STUDY00008079.

Study Inclusion Criteria 

Experienced neonatologists working in the MCP role in neonatal transport were recruited to participate in the lighting assessments. There were no exclusion criteria.

Study procedure

Manikin and Transport Incubator

The study was conducted using a Gaumard Newborn Tory S2220 neonatal manikin with a medium skin tone (Gaumard Scientific, Miami, Florida, USA). The manikin had settings that included simulating breathing with non-moving limbs and breathing with moving limbs. A Microsoft Surface tablet running the Unified Simulator Controlled Software (UNI) was used to control the manikin. The manikin was placed flat on its back in a neonatal transport incubator, the Airborne Voyager Transport Incubator (International Biomedical Austin, Texas, USA). The incubator features head and front access doors, with two hand ports on the front door and back of the infant chamber with a height of 28.9 inches by 19 inches in width and 39.2 inches in length. 

Incubator Lighting

**Figure 1:**
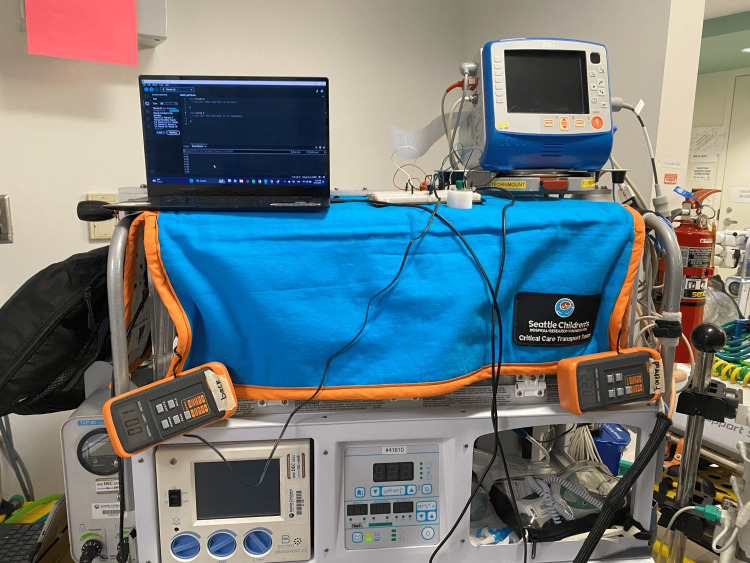
Experimental setup with a laptop running Arduino code controlling the custom dimmer and light meters shown outside of the incubator

A two-and-a-half-foot-long strip of Lepro Dimmable LED tape was attached to the incubator using masking tape in one of two locations: along the inside upper long edge above the access door (upper position) and along the outside lower long edge opposite the access door (lower position). Arduino Integrated Development Environment (Arduino) is an open-source platform that allows for controlling outputs on electronic devices. A custom dimmer was programmed using Arduino, where the light increased or decreased linearly between the measured maximum brightness of 365 lux and being completely off over twenty seconds with 13 bits of resolution. The light controller was operated through serial communication using a Samsung laptop with an Intel Core i5-1135G7 @ 2.40GHz and 8 GB RAM, running Windows 11. This is shown in Figure 1, with the circuit dimmer also displayed on top of the incubator. 

**Figure 2:**
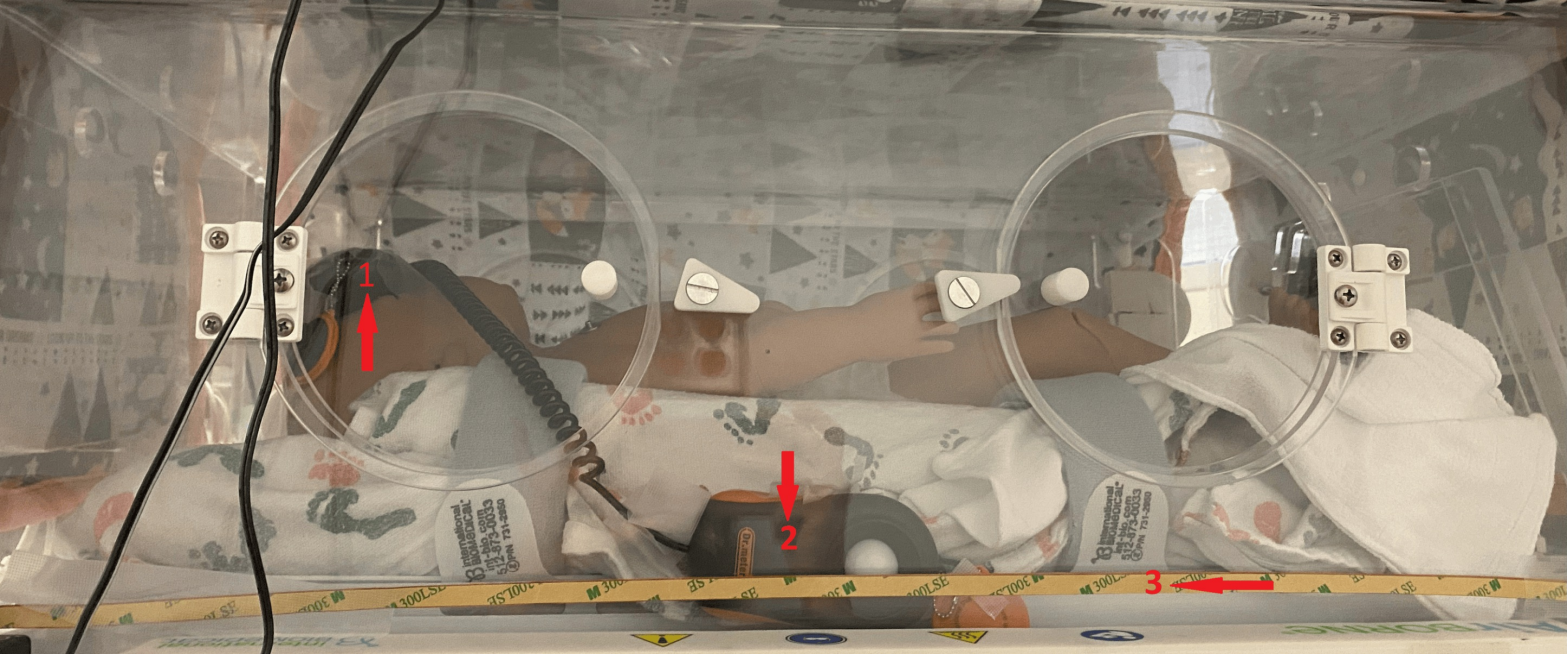
Experimental setup with the light in the lower position showing light strip and light meter positioning, with 1 as the light meter sensor at the forehead, 2 as the light meter sensor measuring the light source, and 3 as the light strip

**Figure 3:**
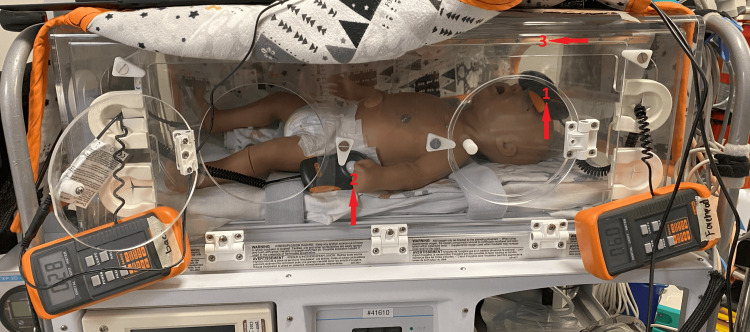
Experimental setup with the light in the upper position showing light strip and light meter positioning, with 1 as the light meter sensor at the forehead, 2 as the light meter sensor measuring the light source, and 3 as the light strip

Two light meters were used to measure light intensity, one placed on the forehead of the manikin and one pointed directly at the light source, as shown in Figures 2, 3. These were Dr.Meter LX1330B Digital Illuminance Light Meters (Dr.meter, Newark, USA). The light sensors would be taped in place using masking tape during the procedure. The light meter displays were hung outside of the incubator through the tube ports to be readable without introducing additional light into the incubator. The ambient light inside the incubator was measured to be 1.4 lux. A phone camera was taped on top of the incubator, beneath the cover, so that the manikin’s face and chest were in view, to record the manikin inside the incubator. A second phone was set such that it captured the view outside of the incubator of both light meter screens. Both phones were Samsung Galaxy S9s. The camera specs had a pixel size of 1.4 µm and used a 12MP AF sensor. 

To mimic transport conditions, a cover was placed over the incubator during the procedure, as previously shown in Figure 1. The atmospheric light level outside the incubator was measured by a light meter to be 83 lux outside the incubator's main door.

Views of Manikin in Moving and Non-moving States

Videos were captured of a breathing manikin in a limp state with non-moving limbs and of a breathing manikin with moving limbs. The phone cameras each captured one long video, covering all the experimental rounds. Before each replication, an experimenter clapped their hands loudly to help with synchronization between the video recordings. The recordings were edited to include time stamps and clipped to be identical in length and synchronized. The manikin video was split into multiple clips, each of a single replication. The light meter video was split into the same frames.

MCP Assessments

Twelve video clips showing views of the manikin inside the incubator under different light conditions were put in a survey (for details of videos and the survey, see the Appendices). These twelve videos were chosen out of thirteen total videos obtained due to the clarity of the neonate, completeness of the video, and ability to accurately view the light meter reading. The videos varied in light position (ten with the light in the lower position, two with the light in the upper position), brightness of light (six with decreasing light from 365 to 1.4 lux, six with increasing light from 1.4 to 365 lux), and manikin behavior (seven were non-moving, five were moving). For clips with the light decreasing and increasing, the survey asked participants to designate the timestamp where they felt they stopped being able to see the manikin well enough to provide proper care. This survey was distributed to participating MCPs by email. Visibility is defined relative to when the provider can see the patient well enough to provide care. The time stamps indicated in the survey responses correlated to times in the corresponding light meter clips to provide light intensity measurement values. Plots were generated using Microsoft Excel (Microsoft Corporation, Redmond, USA) to study the distribution and variability of the data. 

Sample size calculation 

This was a pilot study with a convenience sample of eight neonatologists.

Statistical analysis 

The coefficient of variation was calculated using Microsoft Excel in order to understand the dispersion of the data values around the mean value for each rater for experimental conditions where multiple rounds were presented to the MCPs. The variation in each MCP’s values reported is calculated using the coefficient of variation, as the ratio of the population standard deviation to the mean, and expressed as a percentage. 

## Results

A total of eight MCPs participated in this study. Participant demographics varied with age and years of experience in overseeing neonatal transport. Most participants were female (6; 75%) and aged 36-50 years (6; 75%) (Table 1). The frequency of transport oversight and years of experience varied from minimal to extensive.

**Table 1:** Participant demographics

Demographics (n=8)	n (%)
Sex	
Female	6 (75%)
Male	2 (25%)
Age	
36-50	6 (75%)
51-65	2 (25%)
Years overseeing neonatal transport	
1-5	2 (25%)
6-10	4 (50%)
20+	2 (25%)
Average number of neonatal transports overseen each month	
0-5	3 (37%)
6-10	4 (50%)
10-15	1 (13%)

**Figure 4:**
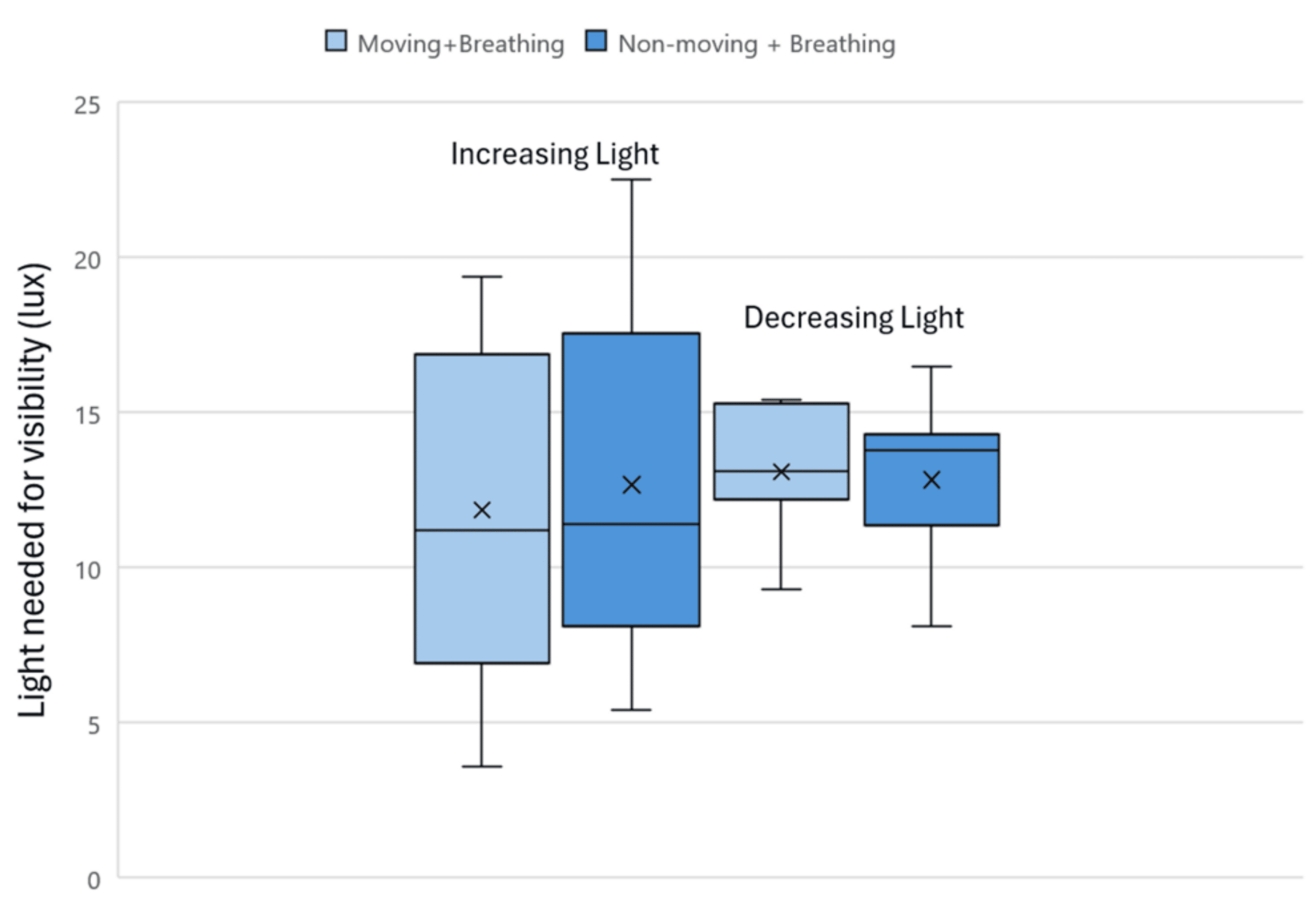
Light needed for visibility of the patient with light in the lower position measured at the forehead for increasing and decreasing light

When the light source was in the lower position in the incubator, the minimum amount of light needed for visibility, as measured at the forehead of the manikin and rated by participants, varied as the light intensity increased or decreased (Figure 4). The range of light needed for visibility in increasing light when the manikin was moving was 3.6-19.4 lux. In decreasing light, the range of light needed for visibility with a moving manikin was 9.3-15.4 lux. When the manikin was non-moving, the range needed for visibility in increasing light was 5.4-22.5 lux and 8.1-16.5 lux in decreasing light. The significance, as calculated through a paired t-test, measured p=0.41 for the moving and breathing condition and p=0.88 for the non-moving and breathing condition.

**Figure 5:**
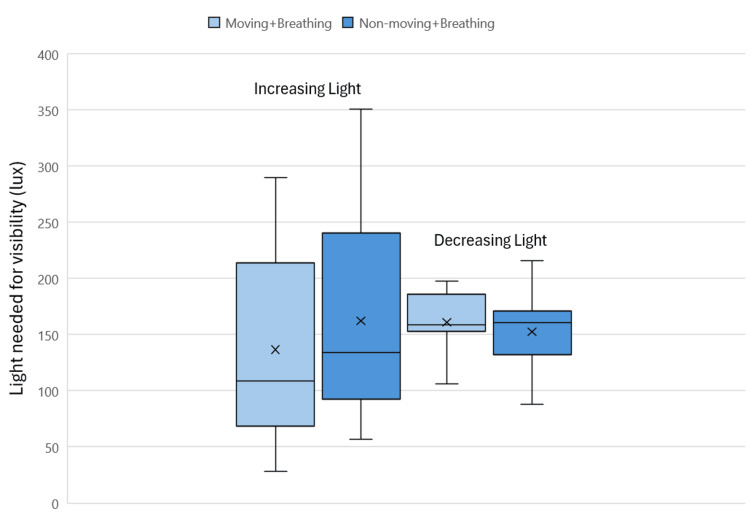
Light needed for visibility of the patient with light in the lower position measured at the light source for increasing and decreasing light

When measured at the light source with the light in the lower position in the incubator, the minimum amount of light needed for visibility as rated by participants varied with the movement of the manikin (Figure 5). The range of light needed for visibility in increasing light when the manikin was moving was 28-290 lux, as measured at the light source. In decreasing light, the range of light needed for visibility with a moving manikin was 106-198 lux. When the manikin was non-moving, the range needed for visibility in increasing light was 57-351 lux and 88-216 lux in decreasing light. The significance, as calculated through a paired t-test, measured p=0.30 for the moving and breathing condition, and p=0.59 for the non-moving and breathing condition. 

When looking at the data for the minimum amount of light needed for the visibility of neonates with the light bar in the lower position and measured as the light increases, the greatest coefficient of variation values were for the moving and breathing patient condition. For the moving and breathing conditions, the coefficient of variation was 6.5% and 16.7% greater for the forehead and direct light meter measurements respectively, when compared to that of the non-moving and breathing conditions.

With the light bar in the lower position as the light decreased, the greatest coefficient of variation values were for the non-moving and breathing patient condition. The coefficient of variation was 1.9% and 1% greater for the forehead and direct light meter readings respectively for the non-moving and breathing condition compared to the moving and breathing condition.

**Figure 6:**
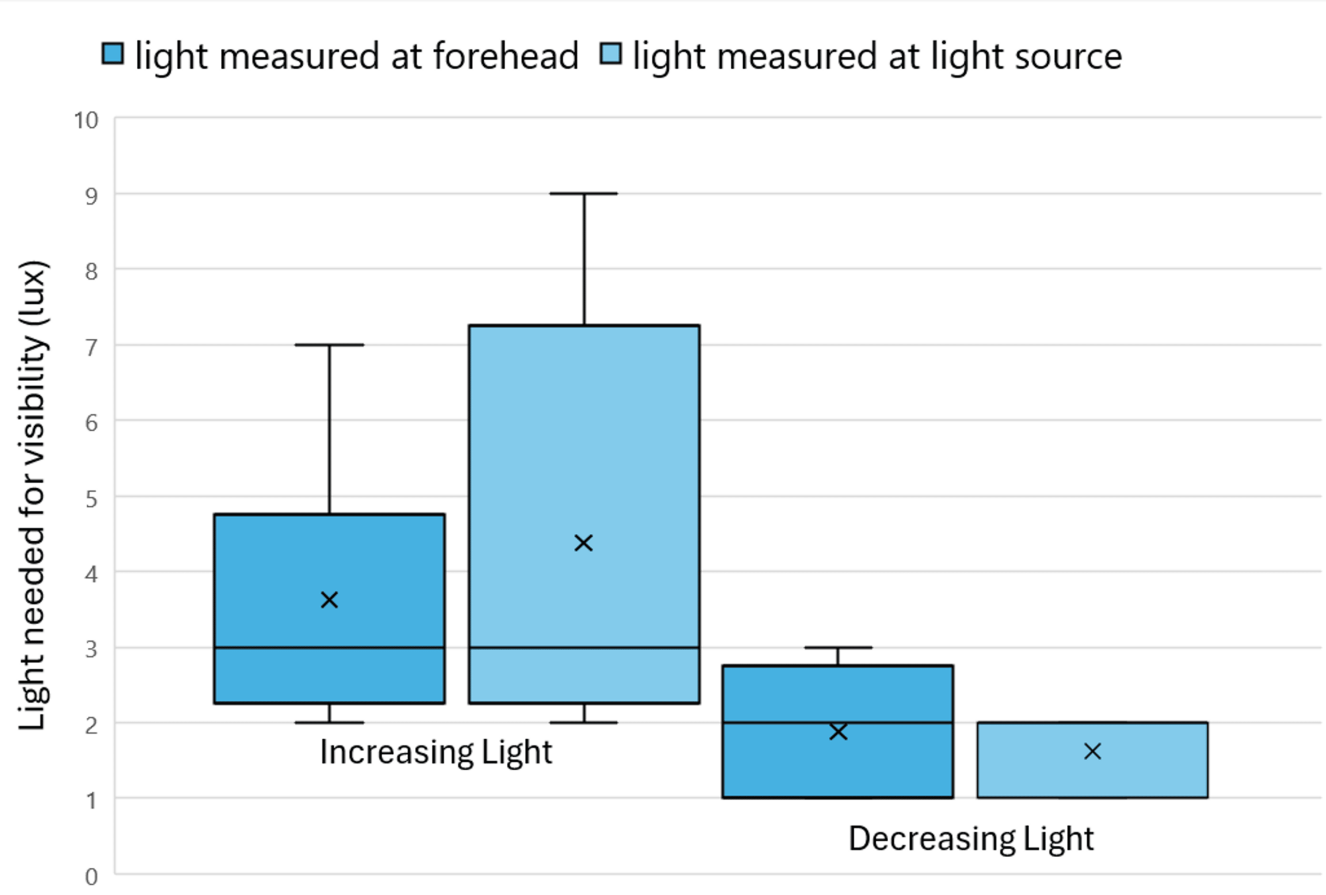
Light is needed for the visibility of non-moving patients with light in the upper position

When the light source was in the upper position in the incubator, the minimum amount of light needed for visibility, as measured at the forehead of the manikin and light source, varied as the light intensity increased or decreased (Figure 6). The range of light needed for visibility in increasing light measured at both the forehead and the light source was greater in increasing light than in decreasing light. When the manikin was non-moving, the range of light needed for visibility in increasing light was 2-9 lux and 1-3 lux in decreasing light.

## Discussion

This is one of the first studies to describe light levels in a simulated neonatal transport setting. We found that the light needed for the visibility of the patient ranged from 1 to 22.5 lux at the forehead and 1 to 351 lux at the light source. However, using the direct light measurement would be the safest approach since bright light can negatively impact the neonate’s eye development [7]. The location of the light source for optimal viewing of the simulated patient was in the upper position, with better visibility of the neonate with less light. More light was needed when the light intensity was increasing than when it was decreasing. As the visibility of the patient by a remote MCP is limited by the amount of light in the transport incubator, these findings are relevant to the use of video for patient monitoring during transport [8].

There was variability in the minimum amount of light by location of the light source in relation to the manikin with the best ratings received when the light source was located above the primary access door. This corresponds to the light source that was closest to the manikin. Transport incubators have light sources that are located at the front, top, or back of the incubator or both [9]. Newer transport incubators with light sources at the front or top of the incubator may provide better options for monitoring the infant’s status [9]. The light needed for the visibility of the patient when the light is in the upper position is greater in increasing light conditions than when light is decreasing steadily.

At the beginning of the experimental rounds where light was increasing, the manikin’s movement and position were not visible. As the light intensity slowly increased, the manikin gradually came into view. Conversely, for the experimental rounds where the light decreased, the manikin was clearly visible to participants. This view might have influenced their understanding of the patient’s condition even as the visibility diminished. Therefore, the increasing light condition may be a better representation of clinical situations of the minimum amount of light required from the MCP’s perspective when viewing a patient for the first time.

In the context of neonatal transport, light safety is an important consideration with regard to light exposure to the neonate’s developing eyes. It is therefore critical to consider the safety of the light used. Expert recommendations for examining the neonatal patient are that the exams should be done in a range of 10-600 lux light [10]. However, exams are administered using short-term periods of light and conditions are different from continuous monitoring. There will be no light in the neonatal transport incubator unless something is detected and further monitoring is needed. All the light values designated by the neonatologists as the minimal amount of light needed were within this range. Specifically, the greatest amount of light designated with the light source in the back of the incubator was about 351 lux, and when the light was above the primary access door, the minimal amount of light was 9 lux at its greatest. Therefore, the light used was safe according to these guidelines. Further studies will be needed to determine the light safety for continuous versus intermittent monitoring during transport. 

The minimum amount of light needed was measured to be higher for the non-moving manikin. This may be because the moving manikin’s chest and limbs may have had more motion artifacts, decreasing the light level that was considered by the MCPs to be adequate for clear visibility of the manikin’s breathing and limb movements. Previous studies support this, having found that changes in the amount of light, including environmental light, impacted the visibility of the camera to focus on the neonate inside the incubator, as the camera needs light to focus on the neonate [11]. Motion, especially variations in the neonate’s movement, has been demonstrated in other studies to impact the amount of light required for visibility [12]. Of note, there was lower variation in the minimum visibility ratings when the light intensity decreased for the moving and breathing manikin. Additionally, skin color may have an impact on visibility, such as impacting the amount of discoloration of the skin a physician can view. However, this study only used one manikin of medium skin tone, and this limitation was not studied. These findings have clinical implications for clinical teletransport applications with moving patients, such as impacting accuracy and MCP visibility of the patient. 

One limitation of this study is the number of measurements conducted. There were two or three rounds for each set of experimental conditions; however, some video footage was lost by the recording device, which limited the number of video samples that could be incorporated in the survey. Another limitation is the number of participants who reviewed the videos. A greater number of participants may have decreased the variability in the results. Additionally, participants used their own devices to view the videos. While this would occur in actual teletransport monitoring, the difference in the preferred brightness of their devices and the device model may have impacted their responses. Therefore, MCP viewing devices changed the brightness of the video viewed, and this was not taken into account. This was done because during the monitoring of neonatal transports, MCPs are each in different environments when viewing the baby and use their own personal devices. 

## Conclusions

This study looked at lighting inside neonatal incubators with implications for neonatal transport with remote monitoring. The minimal amount of light needed for the visibility of the neonatal patient was relatively low compared to the recommended light thresholds for safety. When the light source was above the primary access door of the incubator, less light was needed. Responses varied between participants, indicating that different physicians have differing opinions on the amount of light needed to effectively view the patient. Future studies should obtain additional measures and include a greater number of participants to better define the light intensity required for the visibility of the patients during neonatal transport. 

## Appendices

For details of views of the manikin inside the incubator under different light conditions and the survey, see https://drive.google.com/drive/folders/1UYc3q1pN1iSwzu1IMmIxQAyV4JCPGNMS?usp=sharing
